# Andrographolide, an Anti-Inflammatory Multitarget Drug: All Roads Lead to Cellular Metabolism

**DOI:** 10.3390/molecules26010005

**Published:** 2020-12-22

**Authors:** Rafael Agustín Burgos, Pablo Alarcón, John Quiroga, Carolina Manosalva, Juan Hancke

**Affiliations:** 1 Laboratory of Inflammation Pharmacology, Faculty of Veterinary Sciences, Institute of Pharmacology and Morphophysiology, Universidad Austral de Chile, Valdivia 5090000, Chile; pabloalarcon.u@gmail.com (P.A.); john.quiroga@uach.cl (J.Q.); juan@hpingredients.com (J.H.); 2 Laboratory of Immunometabolism, Institute of Pharmacology and Morphophysiology, Faculty of Veterinary Sciences, Universidad Austral de Chile, Valdivia 5090000, Chile; 3 PhD Program in Veterinary Sciences, Faculty of Veterinary Sciences, Universidad Austral de Chile, Valdivia 5090000, Chile; 4 Faculty of Sciences, Institute of Pharmacy, Universidad Austral de Chile, Valdivia 5090000, Chile; carolinamanosalva@uach.cl

**Keywords:** andrographolide, anti-inflammatory, multitarget, metabolism, glycolysis

## Abstract

Andrographolide is a labdane diterpene and the main active ingredient isolated from the herb *Andrographis paniculata*. Andrographolide possesses diverse biological effects including anti-inflammatory, antioxidant, and antineoplastic properties. Clinical studies have demonstrated that andrographolide could be useful in therapy for a wide range of diseases such as osteoarthritis, upper respiratory diseases, and multiple sclerosis. Several targets are described for andrographolide, including the interference of transcription factors NF-κB, AP-1, and HIF-1 and signaling pathways such as PI3K/Akt, MAPK, and JAK/STAT. In addition, an increase in the Nrf2 (nuclear factor erythroid 2–related factor 2) signaling pathway also supports its antioxidant and anti-inflammatory properties. However, this scenario could be more complex since recent evidence suggests that andrographolide targets can modulate glucose metabolism. The metabolic effect of andrographolide might be the key to explaining the diverse therapeutic effects described in preclinical and clinical studies. This review discusses some of the most recent evidence about the anti-inflammatory and metabolic effects of andrographolide.

## 1. Introduction

*Andrographis paniculata* is a traditional herb from Asian countries that has been used for relieving and reducing the severity and duration of symptoms of common colds and alleviating fever, coughs, and sore throats in uncomplicated respiratory tract infections. Over the last decades, many Asian and European researchers have begun to investigate the composition, activity, safety, and efficacy of this ancient herb, confirming that the traditional medicine has a sound basis. The biological effects of *A. paniculata* are related to the constituents of its aerial parts, a group of diterpene lactones belonging to the ent-labdane class, present in both free and glycosidic forms. The main constituent of *A. paniculata* is andrographolide, a diterpene that contains a γ-lactone ring that is present in leaves in quantities varying from 51.22 ± 0.04 to 68.35 ± 1.50 mg/g [1]. Other diterpenes such as 14-deoxy-11, 12-didehydroandrographolide, and neoandrographolide varied from 5.38 ± 0.30 to 16.01 ± 0.8 mg/g and 5.47 ± 0.03 to 11.72 ± 0.33 mg/g, respectively. Meanwhile, 14-deoxyandrographolide shows high levels in leaves only at the transfer stage in field experiments (30.59 ± 1.39 mg/g) [1].

Methanolic and aqueous extracts of *A. paniculata*, or andrographolide, exhibited an antioxidant and acute anti-inflammatory effect against carrageenan-induced paw edema in rodents [2], suggesting that andrographolide may be responsible for the pharmacological properties described for the herb in traditional medicine [3]. In addition, andrographolide shows the most potent anti-inflammatory [4] and anticancer activities, with stronger effects than 4-deoxy- 11,12-didehydroandrographolide and 14-deoxyandrographolide [5].

**Figure 1:**
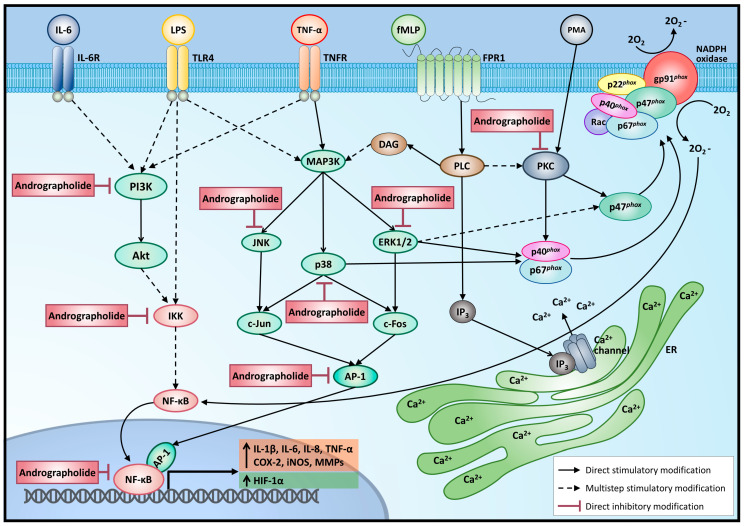
Andrographolide exerts anti-inflammatory effects by inhibiting several intracellular signaling pathways activated by different pro-inflammatory agents. Akt = protein kinase B; AP-1 = activator protein 1; Ca^+2^ = calcium ion; COX-2 = cyclooxygenase 2; DAG = diacylglycerol; ERK1/2 = extracellular signal-regulated kinases 1/2; fMLP = N-Formyl-methionyl-leucyl-phenylalanine; FPR1 = formyl peptide receptor 1; HIF-1α = hypoxia inducible factor 1, alpha subunit; IKK = I-kappa-B kinase; IL-1β = interleukin 1 beta; IL-6 = interleukin 6; IL-6R = IL-6 receptor; IL-8 = interleukin 8; iNOS = inducible nitric oxide synthase; IP3 = inositol triphosphate; JNK = c-Jun N-terminal kinase; LPS = lipopolysaccharide; MAP3K = mitogen activated protein kinase kinase kinase; MMPs = matrix metalloproteinases; NADPH oxidase = nicotinamide adenine dinucleotide phosphate oxidase complex; NF-κB = nuclear factor kapa B; PI3K = phosphatidyl inositol 3 kinase; PKC = protein kinase C; PLC = phospholipase C; PMA = phorbol myristate acetate; TLR-4 = Toll-like receptor 4; TNF-α = tumoral necrosis factor alpha; TNFR = TNF-α receptor.

Andrographolide’s antioxidant properties have been related to its anti-inflammatory mechanism [6]. In fact, together with nitric oxide, reactive oxygen species (ROS) like hydrogen peroxide H_2_O_2_ or superoxide anion O_2_^−^ are involved in the intracellular killing of microorganisms. In this way, the release of extracellular DNA traps by neutrophils, a process regulated by the mitogen-activated protein kinase (MAPK) pathway that involves the extrusion of DNA decorated with cytoplasmic proteins and with antimicrobial and proteolytic activity, is a defense mechanism of neutrophil and other leukocytes against infectious processes or pro-inflammatory agents [7]. Indeed, in neutrophils, several proinflammatory stimuli induce the ROS-dependent formation of neutrophil extracellular traps (NETs) [7], including microorganisms and proinflammatory factors [8]. However, because of their uncontrolled production by phagocytes during the inflammation process, they may become toxic metabolites, leading to severe tissue damage in several pathologies. For example, excessive ROS production and NETs release by proinflammatory stimuli plays an important pathological factor in the induction of ischemic and reperfusion injury, metabolic impairments, and liver diseases [9,10]. In rat neutrophils, pretreatment with andrographolide prevented phorbol-12‒myristate-13‒acetate (PMA)- induced ROS production and accumulation, as well as N-formyl-methionyl-leucyl-phenylalanine (fMLP)-induced adhesion and transmigration [11]. Andrographolide inhibited the upregulation of CD11b and CD18 induced by fMLP [12]. The overexpression of Mac-1 (Macrophage-1 antigen or CD11b/Cd18, involved in the adhesion of leukocytes onto the endothelium) by neutrophils is regulated by the accumulation of ROS and intracellular calcium [Ca^2+^]_i_ mobilization; andrographolide pretreatment reduced the fMLP-induced production of H_2_O_2_ and O_2_^−^ but did not affect the [Ca^2+^]_i_ mobilization. Andrographolide interferes with the effects of PMA, a direct protein kinase C (PKC) activator, suggesting that an inhibition of ROS production through the modulation of PKC-dependent pathway (Figure 1) could explain, at least in part, the ability of andrographolide to downregulate Mac-1 expression, essential for neutrophil adhesion and transmigration during inflammation [12]. In addition, PMA is a potent NET inducer; in fact, andrographolide alleviates murine arthritis by reduced neutrophil infiltration and NET formation [13].

Andrographolide reduces the expression of several proinflammatory genes, including cyclooxygenase-2 (COX-2), IL-6, IL-8, IL-1β, and inducible nitric oxide synthase (iNOS) in endothelial cells, synoviocytes, colorectal cancer cells, and leukocytes [14]. One milligram per kg b.w. andrographolide administered to rodents by i.v. reduces the pro-inflammatory and hemodynamic effects of lipopolysaccharides (LPS) [15]. In addition, 1 mg/kg i.p. andrographolide protects against LPS-induced acute lung injury, reducing myeloperoxidase (MPO) activity, neutrophils, macrophages, TNF-α, IL-6, and IL-1β in the bronchoalveolar lavage fluid of rodents [16]. In Freund’s adjuvant-induced complete arthritis or collagen-induced arthritis models in rodents, andrographolide (3‒6 mg/kg i.p.) diminishes the clinical score of arthritis and joint damage and reduces the production of NO and TNF-α [17], indicating that andrographolide may be employed as a natural anti-inflammatory or for the synthesis of more potent derivatives [18].

On the other hand, chronic inflammation of tissue or organ is characterized by the presence of inflammatory cells (immune cells) and the proliferation/regeneration of damaged tissue. Sustained enhanced cellular proliferation in an environment with abundant inflammatory cells, growth factors, and DNA damage and/or mutagenic insult certainly potentiates and/or promotes carcinogenesis risk [19,20]. Andrographolide exhibits antineoplastic activities through COX-2 inhibition and reduced proliferation in human breast cancer [21]. Moreover, andrographolide antagonizes TNF-α-induced IL-8 via inhibition of the NADPH oxidase/ROS/NF-κB and Src/MAPKs/AP-1 axes (Figure 1) in human colorectal cancer HCT116 cells and suppresses angiogenesis in the tumor microenvironment [22].

Hypoxia-inducible factor 1 (HIF-1) controls metabolic reprogramming in cancer cells via gene expression of glucose transporters and glycolytic enzymes [23]. In addition, andrographolide reduced hepatoma cancer cells’ growth, inhibiting vascular endothelial growth factor A expression via HIF-1α degradation [24], which also suggests that andrographolide could interfere with cell metabolism in cancer.

It has been demonstrated that an ethanolic extract of *A. paniculata* and andrographolide showed an alpha-glucosidase inhibitory effect in a concentration-dependent manner, supporting a potential use for the management of type 2 diabetes mellitus [25]. Moreover, andrographolide downregulated the expression of sterol regulatory element-binding proteins’ (SREBPs) target genes, decreased cellular lipid accumulation in vitro, ameliorated lipid metabolism, and improved glucose use in mice with high-fat-diet-induced obesity [26]. In addition, andrographolide suppressed the TNF-α-induced activation of the NF-κB signaling pathway and its downstream inflammatory factors’ expression, ameliorating insulin resistance in 3T3-L1 adipocytes [27], which suggests a close relationship between the anti-inflammatory effects and metabolic modulation by andrographolide.

In addition, andrographolide has been useful in the therapy of inflammatory processes and metabolic disorders [14,28]. Both biological properties have been analyzed separately; however, during inflammation, several disturbances of metabolism occur at once. Moreover, these effects could be part of the common signaling pathways modulated by andrographolide.

## 2. Andrographolide Target Involved in Anti-inflammatory and Metabolic Effects

### 2.1. Metabolism and Inflammation

Inflammation involves the coordinated liberation of blood components (plasma and leukocytes) to the injured tissue. The recruitment of leukocytes from the blood, together with tissue-resident immune cells, ensures efficient pathogen killing and contributes to healing. Importantly, the duration and intensity of the inflammatory response is controlled by the secretion of several molecules such as cytokines or lipid mediators and chemokines that recruit innate and adaptive immune cells to the site of injury [29]. A successful inflammatory response is followed by the resolution phase, which gradually reduces the inflammation when the danger signal or the injury has been eliminated; this phase is critical for restoring homoeostasis [30]. Under certain circumstances, the immune cells fail to resolve inflammatory processes, and the inflammation progresses over time to chronic inflammation, which includes several chronic and age-associated human pathologies, such as neurodegenerative, cardiovascular, joint, and muscular diseases [31].

**Figure 2:**
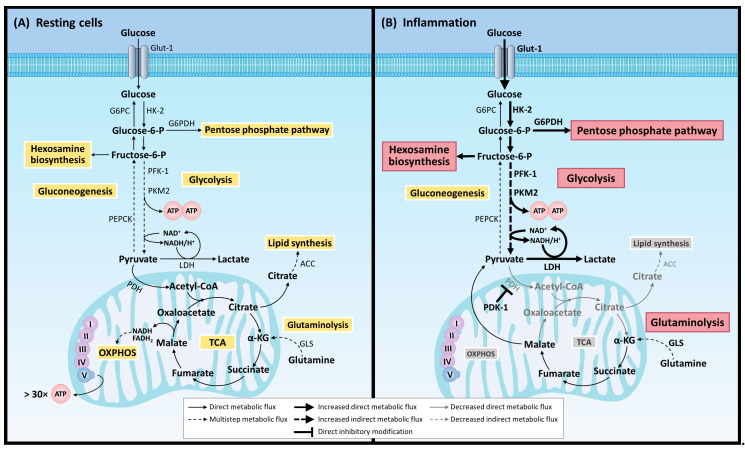
Predominant metabolic pathways activated in resting cells and during inflammation. ACC = acetyl-CoA carboxylase; ATP = adenosine triphosphate; α-KG = alpha-ketoglutarate;FADH_2_ = reduced flavin adenine dinucleotide; GLS = glutaminase; Glut-1 = glucose transporter 1; G6PC = glucose-6-phosphatase catalytic subunit; G6PDH = glucose-6-phosphate dehydrogenase; HK-2 = hexokinase 2; LDH = lactate dehydrogenase; NAD^+^ = oxidized nicotinamide adenine dinucleotide; NADH/H^+^ = reduced nicotinamide adenine dinucleotide; OXPHOS = oxidative phosphorylation; PDH = pyruvate dehydrogenase; PDK-1 = pyruvate dehydrogenase kinase 1; PEPCK = phosphoenolpyruvate carboxykinase; PFK-1 = phosphofructokinase 1; PKM2 = pyruvate kinase M2 isoform; TCA = tricarboxylic acid cycle.

Cells use macromolecules that are degraded to obtain the necessary energy for essential functions. Metabolism is a process fundamental to cellular biology, providing energy and building blocks for macromolecule synthesis. One of the main metabolic pathways that provide energy for all cellular processes is glycolysis (Figure 2), which comprises multiple enzymes that include glucose uptake and its metabolization, generating two net molecules of ATP and NADH. Glycolysis consists of 10 consecutive enzymatic reactions that convert glucose into two molecules of pyruvate, which connect with other metabolic pathways. In general terms, there are three rate-limiting/rate-controlling enzymes of glycolysis: hexokinase, phosphofructokinase, and pyruvate kinase (Figure 2).

Glycolysis is usually carried out under hypoxic conditions and is only effective as an energetic route during intense and short exercise of less than 2 min. Furthermore, the accumulation of lactic acid, a by-product of anaerobic metabolic, causes tissue pain and fatigue during exercise and inflammation (Figure 2). While ATP is primarily the energy source for metabolic work, NADH provides reducing power in anabolic reactions and can also be oxidized in the respiratory chain. Thus, the generation of glycolytic ATP and NADH serves as an energy source for aerobic respiration and anaerobic fermentation. The pyruvate generated in glycolysis has access to the mitochondria and fully metabolizes to CO_2_ through the tricarboxylic acid (TCA) cycle, generating NADH and reducing FADH2 to perform oxidative phosphorylation (OXPHOS) (Figure 2). However, pyruvate can also be fermented to lactate under normoxic conditions, regenerating NAD^+^ without generating ATP in a process called aerobic glycolysis. Despite the higher energy efficiency of OXPHOS, highly proliferating cells such as tumor cells prioritize the glycolytic pathway [32]. In fact, cancer cells rewire their metabolism to promote growth, survival, proliferation, and long-term maintenance. The common feature is an increase in glucose uptake and fermentation of glucose to lactate. This metabolic profile is observed even in the presence of oxygen and fully functioning mitochondria; this together is known as the Warburg effect.

The different cell types of the immune system use different metabolic pathways to maintain a good source of energy that allows them a fast and adequate response to an infection or cellular injury. During the inflammatory response, the activation of immune cells also depends on a process of metabolic reprogramming. Thus, while glycolysis is the main energy source at the peak of inflammation, during the resolution phase the immune cells rely mainly on OXPHOS. Therefore, glycolytic metabolism seems to be a new target for the development of anti-inflammatory and antineoplastic drugs [32,33]. The production and influx of neutrophils into the damaged tissue is an earlier event during the acute inflammatory cascade. Mature neutrophils are mainly glycolytic; some examples have shown that mitochondrial respiration and ATP synthesis/release are also important for neutrophil transmigration [34], ROS production [35], and NET formation [36,37]. Moreover, the release of extracellular ROS is mediated by the action of the membrane-bound enzyme NADPH oxidase. The interference of glycolysis led to reduced NADPH oxidase function, NETs formation, and deficient microbial killing [38].

The glycolytic switch is a key for immune cells during inflammation, e.g., pro-inflammatory macrophages (M1 phenotype) or neutrophils. Glycolysis supplies metabolic intermediates for other biosynthetic pathways necessary for cellular growth and differentiation that decrease respiration and a broken Krebs cycle, leading to an accumulation of both citrate and succinate [39]. In addition to glycolysis, the pentose phosphate pathway (PPP), the hexosamine pathway, and glutaminolysis increase upon activation (Figure 2) [38]. On the contrary, M2 macrophages that serve to modulate inflammation, promote tissue repair, and regulate adaptive immunity, the Krebs cycle, and oxidative phosphorylation are intact, and high levels of fatty acid oxidation (FAO) are observed [39].

In T cells, metabolism determinates the quiescent and activated form of naïve T cells. In quiescent T cells, glucose and amino acid catabolism are predominant; however, when T cells exit the naïve state for activation by immunological cues, this induces a metabolic reprogramming (catabolism and anabolism) that allows for increased consumption of extracellular nutrients for the biosynthesis of energy and ATP production by glycolysis or OXPHOS, coupled to the TCA cycle and fatty acid synthesis [39].

The accumulation of several cellular metabolites determines the activation/repression of signaling pathways, the epigenetic and post-transcriptional regulation of inflammatory genes, and the post-translational modification of proteins [39]. Several of these pathways induced in the inflammatory process also control glycolytic metabolism and could contribute to the ability of andrographolide to reduce inflammation and cancer progression [14].

### 2.2. Hypoxia-Inducible Factor 1α (HIF-1α)

**Figure 3:**
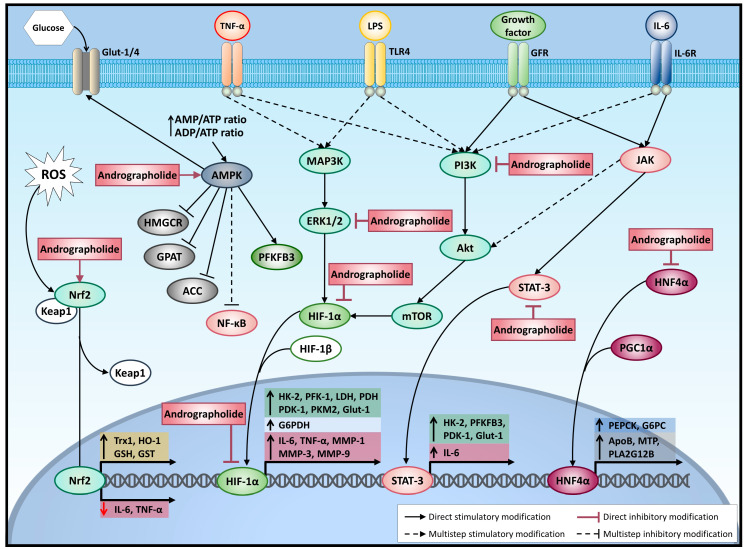
Andrographolide modulates at different levels the metabolic responses induced by proinflammatory agents and growth factors. ACC = acetyl-CoA carboxylase; ADP = adenosine diphosphate; Akt = protein kinase B; AMP = adenosine monophosphate; AMPK = adenosine monophosphate-activated protein kinase; ApoB = apolipoprotein B; ATP = adenosine triphosphate; ERK1/2 = extracellular signal-regulated kinases 1/2; GFR = growth factor transporter; Glut-1/4 = glucose transporter 1/4; GPAT = glycerol-3-phosphate acyltransferase; GSH = Glutathione; GST = Glutathione S-transferase; G6PC = glucose-6-phosphatase catalytic subunit; G6PDH = glucose-6-phosphate dehydrogenase; HIF-1α = hypoxia inducible factor 1, alpha subunit; HIF-1β = hypoxia inducible factor 1, beta subunit; HMGCR = hydroxymethylglutaryl CoA reductase; HK-2 = hexokinase 2; HNF4α = hepatocyte nuclear factor 4 alpha; HO-1 = Heme oxygenase-1; IL-6 = interleukin 6; IL-6R = IL-6 receptor; JAK = Janus kinase; Keap1 = Kelch-like ECH-associated protein 1; LDH = lactate dehydrogenase; LPS = lipopolysaccharide; MAP3K = mitogen activated protein kinase kinase kinase; MMP-1 = matrix metalloproteinase 1; MMP-3 = matrix metalloproteinase 3; MMP-9 = matrix metalloproteinase 9; mTOR = mammalian target of rapamycin; MTP = microsomal triglyceride transfer protein; Nrf2 = nuclear factor erythroid 2–related factor 2; PDH = pyruvate dehydrogenase; PDK-1 = pyruvate dehydrogenase kinase 1; PEPCK = phosphoenolpyruvate carboxykinase; PFK-1 = phosphofructokinase 1; PFKFB3 = 6-phosphofructo-2-kinase/fructose-2,6-biphosphatase; PGC1α = peroxisome proliferator-activated receptor gamma coactivator 1 alpha; PI3K = phosphatidyl inositol 3 kinase; PKM2 = pyruvate kinase M2 isoform; PLA2G12B = phospholipase A2 G12B; ROS = reactive oxygen species; STAT-3 = signal transducer and activator of transcription 3; TLR-4 = Toll-like receptor 4; TNF-α = tumoral necrosis factor alpha; TNFR = TNF-α receptor; Trx1 = Thioredoxin 1.

HIF-1α is a transcription factor controlled by cellular oxygen concentrations; it is easily degraded in normoxia and stabilized in hypoxia. In fact, proline hydroxylases (PHDs), responsible for HIF-1a hydroxylation, or the von Hippel–Lindau protein (*p*VHL) target HIF-1a for ubiquitination and subsequent proteasomal degradation. However, HIF-1α can be sustained even under normoxic conditions by the products of glycolysis, lactate, and pyruvate [40]. At a transcriptional level, bacterial LPS increase the expression of HIF-1α [41]. Induction of HIF-1α mRNA by LPS under normoxic conditions may be of particular importance in inflammatory processes since it prepares monocytes for survival and function in a hypoxic microenvironment before they extravasate from the vasculature into the tissue [42]. In M1 macrophages stimulated with LPS, HIF-1-mediated metabolic reprogramming is dependent on pyruvate kinase M2 (PKM2), a glycolytic enzyme responsible for converting phosphoenolpyruvate (PEP) to pyruvate. While PKM2 tetramers are highly active at a cytosolic level, supporting the final step of glycolysis, PKM2 monomers and dimers lack enzymatic activity [43]. However, PKM2 dimers can translocate to the nucleus and interact directly with HIF-1α, positively regulating the expression of and promotes the expression of downstream target genes [44]. In fact, PKM2 is highly expressed in tumor cells and LPS-stimulated macrophages, and the stabilization of HIF-1α favors the expression of IL-1β and glycolytic enzymes [44]. In addition, HIF-1α controls the glycolytic machinery via the expression of several proteins including hexokinase-2 (HK-2) that catalyze the first step of glucose metabolism: phosphofructokinase-1 (PFK-1), a rate-limiting enzyme of glycolysis; glucose-6-phosphate-dehydrogenase (G6PDH), which catalyzes the first step in the pentose phosphate pathway; lactate dehydrogenase; pyruvate dehydrogenase kinase-1, which inactivates the TCA cycle enzyme; pyruvate dehydrogenase (PDH), which converts pyruvate to acetyl-CoA; and the glucose transporter-1 (GLUT-1) (Figure 3) [45].

HIF-1α acts as a key regulator during inflammation, increasing pro-inflammatory cytokines (e.g., TNF-α, IL-1β, IL-6, and IL-8), matrix metalloproteinases (e.g., MMP-1, MMP-3, and MMP-9) (Figure 3), and pathways of glucose metabolism in rheumatoid arthritis (RA) [46].

Andrographolide reduces MMP-1, MMP-3, and MMP-9 expression of rheumatoid arthritis fibroblast-like synoviocytes (RA-FLS) in hypoxia, decreasing HIF-1α expression and interfering with HIF-1α binding to DNA (Figure 3) [47]. RA-FLS shows a shift from oxidative phosphorylation to glycolytic ATP production; in addition, synovial tissue is enriched in HIF-1α, key to RA pathogenesis [48,49]. In synovial tissue from RA or chondrocytes from osteoarthritis (OA), patients show an increase in PKM2 expression, suggesting a potential new therapeutical target [50,51]. By using a quantitative chemical proteomic approach, novel potential targets of andrographolide have been proposed in the fields of inflammation and immunity research, including PKM2 and LDH [52]. This suggests that andrographolide could reduce the glycolytic switch induced during the joint inflammatory process.

In human clinical trials, patients with RA or moderate knee osteoarthritis treated with 300 mg daily of a standardized dried extract of *A. paniculata* (ParActin^®^: ~150 mg daily of andrographolide) for 16 weeks or one year had reduced swelling joint and pain [53,54]. However, whether this also modulates glucose metabolism is uncertain and should be carefully considered in future studies.

Andrographolide reduces the expression of HK-2, with GLUT-1 reducing glycolysis in human chondrosarcoma SW 1353 cells [55]. Furthermore, andrographolide has been used to develop new anti-inflammatory derivatives that simultaneously show HK-2 inhibition, reducing glycolysis and interfering with the NF-kB pathway, and iNOS and COX2 expression [56]. It has been reported that andrographolide reduces HIF-1α protein and expression through the inhibition of PI3K/Akt-mTOR in breast cancer cells (Figure 3) [57]. In HCT116 colon cancer cells, the inhibition of PI3K/Akt-mTOR–HIF-1α reduces lactate and ATP concentration and GLUT1, HK-2 and PFK-1 expression, supporting the idea that the inhibition of glycolysis is critical for reducing viability [58]. Andrographolide inhibits the PI3K/Akt pathway in human umbilical vein endothelial cells, reducing IL-6, TNF-α, and IL-1β induced by exposure to high glucose [59]. Furthermore, in endothelial cells, andrographolide abolishes TNF-α-induced Akt phosphorylation (Figure 3), decreasing the expression of adhesion molecules such as ICAM-1 [60]. On the other hand, in 3T3-L1 fibroblasts, andrographolide increases glucose uptake, activating the PI3K pathway and restoring the insulin sensitivity impaired by TNF-α [27]. In spite of this, andrographolide reduces the expression of IL-6, iNOS, SOCS3, and MCP-1 induced by TNF-α in 3T3-L1 cells; this is attributable to the inhibition of NF-κB [27].

### 2.3. Nuclear Factor Kappa B (NF-κB) Pathway

NF-κB is a key transcription factor for pro-inflammatory genes’ expression [61]. Activation of NF-κB involves the phosphorylation of the specific inhibitory factor IκBα, by I-kappa kinase (IKK), and phosphorylated IκBα is rapidly degraded by a proteasome, allowing the resultant free NF-κB heterodimer (*p* 50/*p* 65) to translocate into the nucleus and induce gene transcription (Figure 1) [61]. The analysis of the HIF-1α promoter showed binding sites for NF-κB. Moreover, inhibition of NF-kB reduced the LPS-induced increase in HIF-1α mRNA but also reduced constitutive HIF-1α expression [42], suggesting that proinflammatory stimuli could alter the cellular metabolism.

Andrographolide interferes with NF-κB binding to DNA in neutrophils, thus reducing the expression of proinflammatory proteins. Mechanistically, andrographolide formed a covalent adduct with the reduced cysteine (62) of *p* 50-NF-κB, thus blocking the binding to DNA and reducing transcriptional activity (Figure 1) [14]. Moreover, other authors found that andrographolide inhibits TNF-α-induced IKK activation and IκBα phosphorylation in endothelial cells [62]. This anti-inflammatory property has been associated with its efficacy in ameliorating ulcerative colitis, asthma, hepatic inflammation, and arthritis [14,17].

In addition, NF-κB affects carbohydrate metabolism by increasing HIF-1α expression. Proinflammatory cytokines and short-term hypoxia lead to phosphorylation of IκB, thus releasing NF-κB, which binds to the promoter of the HIF-1α gene, thereby increasing HIF-1α mRNA and protein levels [63]. The inhibition of NF-κB by p65 siRNA or BAY 11–7082 in RA-FLS led to a marked decrease in the HIF-1α, MMP2, and MMP9 expression induced by IL-17A [46]. This supports the interplay between inflammation and metabolism, a hallmark observed during joint inflammation. Moreover, Walmsley et al. [64] demonstrated a close relationship between HIF-1 and NF-κB in neutrophils. In fact, the NF-κB p65 subunit and IκBα regulator IKKα were identified as HIF-1 target genes, indicating that NF-κB is an important downstream regulator of the hypoxic response in neutrophils [64]. These observations further support the notion that HIF-1 and NF-κB work in cooperation in inflammatory conditions and in hypoxia. In fact, there is a relationship between NF-κB and tumor progression-associated inflammation that controls the expression of cancer-associated genes such as cytokines, chemokines, growth factors, and transcription factors such as HIF-1α [65]. Andrographolide suppresses the proliferation of human cancer colon and multiple myeloma cells via TLR4/NF-κB inhibition [66].

### 2.4. MAPK-Src and AP-1

Src kinases are a large family of nonreceptor protein tyrosine kinases (PTK) that control multiple signaling pathways, are expressed either ubiquitously or predominantly in specific immune-competent cells, and are involved in a variety of immunologic processes, such as immune cell development, proliferation, adhesion, migration, chemotaxis, phagocytosis, and survival. Src kinases have upstream crosstalk activation with MAPK and phosphatidylinositol 3-kinase (PI3K)/Akt pathways [67]. The activation of tyrosine kinase receptor by growth factor induces activation of the PI3K/Akt pathway, which stimulates glucose uptake via GLUTs and increases glycolysis during cell growth and survival [68]. On the other hand, MAPK pathways are signaling modules that transduce extracellular and intracellular cues via the phosphorylation of key protein targets. After the appropriate cellular cue, successive activation of a MAPK kinase kinase (MAPKKK) and a MAPK kinase (MAPKK) results in the phosphorylation/activation of three main families: ERKs (extracellular-signal-regulated kinases), JNKs (Jun amino-terminal kinases), and *p* 38/SAPKs (stress-activated protein kinases) (Figure 1).

The ERK1/2 pathway is involved in HIF-1α activation through p300/CBP [45]. LPS increased HIF-1α via ERK1/2 in human monocytes as well as in nondifferentiated cells and the differentiated human monocytic cell line THP-1 under normoxic conditions [42], with a reducing effect on metabolism and inflammation. Andrographolide reduces the phosphorylation of MAPK such as ERK1/2, p38, and JNK in LPS-stimulated RAW264.7 cells [69] or TNF-α stimulated RA-FLS [70]. Andrographolide suppressed LPS-induced phosphorylation of ERK1/2, JNK, and *p* 38 in mouse RAW264.7 cells (Figure 1) [69]. MAPK (ERK 1/2 and *p* 38 MAPK) activate the transcription factor activator protein-1 (AP-1). In fact, andrographolide inhibits Src and ERK1/2, interfering with the activation of AP-1 (Figure 1) and antagonizing TNF-α-induced IL-8 in HCT116 human colorectal cancer cells [22].

The AP-1 complex (homo- and heterodimer) is a dimeric transcription factor encompassing a group of structurally and functionally related members of the Jun, Fos, ATF, and MAF protein families. In the immune system, a variety of different cytokines and chemokines are predominantly regulated by AP-1. In addition, increased expression of AP-1 is involved in certain types of cancer such as breast cancer, endometrial carcinoma, and colorectal cancer [71]. Moreover, the inhibition of AP-1 has been related to siRNA attenuated cytokine expression (TNF-α, IL-1β, and IL-6) in macrophages and reduces lipid accumulation in hepatocytes induced by palmitic acid [72], suggesting the involvement of AP-1 in inflammation and hepatic lipid metabolism. Andrographolide reduces the activation of AP-1 (Figure 1) in human colorectal cancer [22] and endothelial cells [73], induced by TNF-α. In addition, andrographolide suppresses the nuclear localization of AP-1 and STAT-1 in macrophages stimulated with LPS, evidence of the inhibitory effect of andrographolide in JAK/STAT pathways (Figure 3) [74].

### 2.5. JAK/STAT Pathway

The Janus kinase (JAK)/signal transducer and activator of transcription (STAT) pathway is activated for several extracellular stimuli such as growth factors (epidermal growth factor receptor (EGFR), granulocyte-macrophage colony-stimulating factors (GM-CSF)), cytokines (IL4, IL6, and IFNγ) [75], and metabolism-relevant hormones (growth hormone, leptin, erythropoietin, and prolactin), leading to critical cellular events such as hematopoiesis, lactation, and the development of the mammary glands and the immune system [75]. Seven mammalian STAT family members have been identified (STAT1, STAT2, STAT3, STAT4, STAT5a, STAT5b, and STAT6) and four JAKs, named JAK1, JAK2, JAK3, and TYK2. The JAK/STAT pathway is associated with autoimmune and inflammatory diseases and cancer [75,76].

Additionally, this pathway is involved in metabolic diseases such as metabolic syndrome, insulin resistance, and obesity-associated metabolic syndrome [75]. The JAK/STAT pathway controls the expression of key glycolytic mediators 6-phosphofructo-2-kinase/fructose-2,6-biphosphatase 3 (PFKFB3), PDK-1, HK-2, glycogen synthase kinase-3α (GSK-3α), and GLUT-1, an effect paralleled by the activation of proinflammatory, proangiogenic, and invasive mechanisms in RA (Figure 3) [77]. Andrographolide reduces IL-6-induced STAT3 phosphorylation and subsequent nuclear translocation in cancer cells (Figure 3) [78]. Moreover, andrographolide suppresses IL-6 cell signaling, including STAT3, ERK, and Akt phosphorylation [79]. In addition, andrographolide reduces the phosphorylation of STAT 1/2, thus interfering with the JAK/STAT pathway in an influenza virus-induced inflammation murine model [80]. In recent years, the JAK/STAT pathway has been implicated in obesity and metabolic syndrome [75]. Indeed, polymorphism of JAK2 is involved in the accumulation of central fat, metabolic syndrome, and lipid metabolic disorder derivates of leptin and insulin activation. Furthermore, polymorphism of STAT5B has been associated with an increase in plasma low-density lipoprotein cholesterol concentrations. Moreover, it has been reported that andrographolide and neoandrographolide reduce the total cholesterol, triglycerides, and low-density lipoprotein cholesterol, suggesting that this molecule has lipidemic effects and could be of help in metabolic syndrome [81]. In addition, STAT3 is essential for normal gluconeogenesis carbohydrate metabolism in the liver [82].

It has been described that andrographolide has a hepatoprotective role. For instance, andrographolide reduces the symptoms of hepatitis [74] and reduces galactosamine and paracetamol-induced liver toxicity [83]. One of the master regulators in the liver, implicated in the metabolism of xenobiotic and metabolic nutrients and waste, is hepatocyte nuclear factor 4 alpha (HNF4α), which has been implicated in drug metabolism, lipid metabolism, and inflammation [84]. In addition, HNF4α possesses a ligand-binding domain that binds with different fatty acids, favoring the interaction with coactivator peroxisome proliferator-activated receptor gamma coactivator 1-alpha (PGC1α) (Figure 3), involved in liver-specific genes related to the metabolism of cholesterol, bile acids, lipids, and glucose [84,85]. In connection with this, andrographolide has been described as a potential HNF4α antagonist, disrupting the interaction with PGC1α (Figure 3). This suppresses the expression of gluconeogenesis enzymes, lowers the plasma glucose level, and ameliorates fatty liver and inflammation in high-fat-diet-fed mice [86]. Moreover, andrographolide reduces RNA replication of the hepatitis C virus in a hepatic cell culture [87] and protects against the acute liver injury induced by LPS and d-galactosamine [88] by activating *p* 38 MAPK pathways and increasing nuclear factor (erythroid-derived-2)-like 2 (Nrf2), which is mediated by the increase in the expression of heme oxygenase-1 (HO-1) (an antioxidant product) [87]. So far, Nrf2 has been implicated in chronic liver damage occurring during viral hepatitis, and in alcoholic and nonalcoholic fatty liver diseases [89].

### 2.6. Nrf2/keap1 Pathway

The Nrf2 and Kelch-like ECH-associated protein 1 (Nrf2/Keap1) pathway is essential for protection against a plethora of diseases that involve inflammation and oxidative stress [90]. Keap1 is one of the key regulators of Nrf2 protein stability. Under normal conditions, Nrf2 is sequestered by Keap1 in the cytoplasm and then degraded by proteasomes, but under stress or oxidative conditions, Keap1 loses the interaction of Nrf2 and translocates to the nucleus to promote the transcription of antioxidant genes (Figure 3) [91]. In addition, oxidative stress stimulates adipose differentiation, which directly triggers obesity and is considered to feed into this pathway; together with the increase in circulating proinflammatory cytokines, this aggravates the insulin resistance in metabolic disorders [92,93]. Beyond antioxidant control, activated Nrf2 also exerts anti-inflammatory effects through the direct inhibition of pro-inflammatory cytokines’ expression, including IL-6 and TNF-α (Figure 3) [94].

Recently, it has been proposed that andrographolide could protect neurons against inflammation-mediated injury via NF-κB inhibition and Nrf2/HO-1 activation. This is attributed to the reduction of NO, TNF-α, and IL-6 release, and decrease in ROS production (Figure 3) in microglia [95]. In addition, andrographolide protects H_2_O_2_ and 6-OHDA-induced oxidative damage in neurons [95].

It has been reported that andrographolide suppresses HIF-1α in human endothelial cell lines, activating Nrf2/HO-1 [96], and reduces the expression of histone deacetylase 1 (HDAC1) [24]. Andrographolide inhibits oxidative biomarkers (such as 8-isoprostane, 8-OHdG, and 3-nitrotyrosine) and increases antioxidant enzyme activity (such as glutathione peroxidase and glutathione reductase) [97]. In this way, andrographolide increases the expression of antioxidant enzymes (such as superoxide dismutase, catalase, glutathione reductase, glutathione peroxidase, glutathione-S-transferase, and reduced glutathione [GSH]) as well as glutathione disulfite (GSSG) concentrations [98]. Andrographolide protects liver cells from oxidative stress via Nrf2/HO-1, probably activating adenosine A2a receptor and reducing the cell death induced by H_2_O_2_ [99]. Additionally, andrographolide increases the redox status in liver cells via downregulation of HNF4A, reducing miR-433 and miR-377 and in this way increasing GSH and HO-1 [100]. In spite of this, the role of the Nrf2/keap1 pathway in acute or chronic liver damage, and tumor development is not completely understood [89], and therefore, these findings should be carefully considered.

### 2.7. AMP-Activated Protein Kinase (AMPK) 

Another important pathway involved in the regulation of Nrf2 in the liver is the AMP-activated protein kinase (AMPK) pathway [101,102]. In mammals, AMPK has been described as the main protein that regulates cellular homeostasis, even acting as a metabolic sensor that is activated when the ratios of AMP/ATP and ADP/ATP have been altered. AMPK is a serine/threonine kinase formed by heterotrimeric protein kinase that is composed of α (two isoforms described), β (two isoforms described), and γ subunits (three isoforms described), where the α subunit is the catalytic one that determines the activity of the protein complex, and the β and γ subunits are the regulatory ones; they are also partly involved in modulation of the kinase complex activity. Activation of AMPK occurs when the AMP level increases, and it binds to the γ subunit, resulting in a conformational change that activates AMPK through phosphorylation at Thr172 in the α subunit by upstream kinases (AMPKK) [103]. However, phosphorylation of AMPKα in Ser485/491, mediated by insulin stimulation, has been shown to lead to a decrease in AMPK activity [104]. Increased activation of AMPK is involved in metabolic reprogramming to switch anabolic metabolism to catabolic metabolism and ignite processes aimed at ATP production or involved in carbohydrate, lipid, and protein metabolism, and is also responsible for the regulation of mitochondrial biogenesis (Figure 3) [103]. AMPK has glycolytic activity through the phosphorylation and activation of PFKFB3 (Figure 3) [105], in the absence of fructose-1,6-bisphosphate (FBP) [106]. Moreover, AMPK regulates carbohydrate metabolism via glucose uptake through GLUT [103]. Andrographolide promotes glucose uptake via GLUT-3 and 4 through the AMPK pathway in rat hippocampal neurons [107]. Four decades ago, AMPK activity was first linked to lipid metabolism. For instance, acetyl-CoA carboxylase (ACC) is an enzyme that can catalyze the carboxylation of acetyl-CoA to malonyl-CoA during the synthesis of fatty acids, or allosterically inhibit CPT-1, a key enzyme in β-oxidation, when AMPK is activated, with inactivated ACC phosphorylating Ser79, Ser1200, and Ser1215 to inhibit fatty acid synthesis and promote β-oxidation [108]. In addition, hydroxymethylglutaryl CoA reductase (HMGCR, a key rate-limiting enzyme in the cholesterol biosynthetic pathway) activation was inhibited by phosphorylation at Ser872 when AMPK was activated (Figure 3) [109]. In this way, the activation of AMPK by AICAR also reduces triglyceride synthesis by inhibition of glycerol-3-phosphate acyltransferase (GPAT) [103,109], evidence of the role AMPK plays in lipid metabolism.

Andrographolide increases AMPK phosphorylation, which reduces the degradation of IκBα and translocation of p65-NF-κB to the nucleus in macrophages [110]. In these cells, an increase in AMPK phosphorylation by andrographolide (10 μM) is associated with an inhibition of MAPK, ERK1/2, p38, and JNK, and a reduction in pro-inflammatory protein expression [110]. Since AMPK is a cellular energy sensor that maintains energy homeostasis via stimulation of glucose uptake and fatty acid oxidation, this could suggest that andrographolide, via AMPK activation, increases the cellular carbohydrate metabolism via OXPHOS and reduces inflammatory processes. In support of this, lactate can reduce Thr172 AMPK phosphorylation in L6 myocytes [111]; in a similar fashion urate crystal, a pro-inflammatory agent in gout, reduces Thr172 AMPK phosphorylation in bone marrow-derived macrophages, which is involved in IL-1β and CXCL1 expression [112]. On the contrary, cell stretching-induced AMPK phosphorylation in rabbit FLS and reduces the NF-κB activation and COX-2 and iNOS expression induced by TNF-α [113]. A reduction of AMPK phosphorylation has been observed in FLS isolated from rats with arthritis; conversely, the glycolysis inhibitor 2-deoxyglucose (2-DG) increases AMPK phosphorylation, interfering with the NF-κB pathway, reducing pro-inflammatory cytokine release [114], and supporting the role of glycolysis in joint inflammation. In addition, andrographolide reduces dextran sulfate sodium-induced acute colitis through AMPK activation [110]. In recent years, andrographolide has been demonstrated to reduce the expression of interferon γ, interleukin (IL)-23, and IL-17A from peripheral blood mononuclear cells derived from ulcerative colitis patients. In these patients, andrographolide had inhibitory effects on Th1/Th17 cells and promoted Th2 cells’ response [115,116]. So far, there have been three clinical trials using *Andrographis paniculata* extract (HMPL-004) for the treatment for ulcerative colitis (NCT00659802 in phase II and NCT01805791 in phase III) and Crohn’s disease (NCT00655733 in phase II). In this respect, an improvement in the clinical severity of ulcerative colitis as assessed by colonoscopy has been observed in patients treated with *Andrographis paniculata* extract [117].

## 3. Conclusions

Cellular metabolism plays a key role in the development of several inflammatory diseases. Andrographolide has been described as a multitarget drug with a myriad of anti-inflammatory, antioxidant, and antineoplastic effects in different cell types. In recent years, andrographolide has been shown to have an effect on cellular metabolism, with potential use in metabolic-related diseases such as diabetes mellitus, hypercholesterolemia, and metabolic syndrome. Moreover, several andrographolide signaling pathway targets participate in glucose metabolism control and could be involved in its effects on inflammation. In addition, the metabolic effect of andrographolide would help explain the miscellaneous therapeutic effects described in preclinical and clinical studies. Therefore, further studies will be required to clarify the relationship between the andrographolide effects on metabolism and inflammation or cancer, which would contribute to a better understanding of its various therapeutic properties and to the development of new derivatives with more selective effects.
